# Decalcifying effects of antimicrobial irrigating solutions on root canal dentin

**DOI:** 10.4317/medoral.18207

**Published:** 2012-08-28

**Authors:** Carmen M. Ferrer-Luque, Mercedes Perez-Heredia, Pilar Baca, María T. Arias-Moliz, María P. González-Rodríguez

**Affiliations:** 1 DDS,MD,PhD. Associate Professor DDS,PhD: Private Practice. DDS,MD, PhD: Professor. DDS, PhD: Associate Professor. Department of Stomatology, School of Dentistry. University of Granada (Spain); 2 DDS, PhD: Assistant Professor. Department of Microbiology, School of Dentistry. University of Granada (Spain); 3 ….

## Abstract

Objective: The aim of this study was to determine the decalcifying efficacy of 7% maleic acid (MA), 2% chlorhexidine (CHX), and combinations of 7% MA + 0.2% cetrimide (CTR) and 2% CHX + 0.2% CTR, in four time periods. Study Design: Four specimens per tooth were obtained from a 2-mm thick slice of the cervical third of the root of ten human incisors. At 1, 2, 3 and 5 minutes of immersion, the concentrations of Ca2+ were measured by atomic absorption spectrophotometry. The results were analyzed using the Mann-Whitney U-test. Results: Statistically significant differences were seen for the extracted calcium in all time periods. The amount of calcium extracted by 7% MA was the highest at all four immersion times, followed by 7% MA + 0.2% CTR. Two percent CHX and its combination with 0.2% CTR extracted virtually no calcium. Conclusions: The decalcifying capacity of 7% MA and 2% CHX diminished when combined with 0.2% CTR.

** Key words:**Cetrimide; chlorhexidine; decalcification; maleic acid; spectrometry.

## Introduction

Chemo-mechanical preparation in endodontic therapy requires the use of antiseptic and chelating agents to eliminate microbes, dissolve organic tissue, and remove the smear layer produced during instrumentation of the root canals (1). Sodium hypochlorite (NaOCl) is used as the main irrigation agent due to its bactericidal power and capacity to dissolve organic matter and necrotic tissue (2,3). Chlorhexidine gluconate (CHX) has been recommended as a root canal irrigant because of its antimicrobial activity, substantivity and low toxicity (4,5).

Chelating and acid solutions are used to remove the inorganic component of the smear layer from the instrumented canals (1,6,7) in time periods of 1 to 10 minutes (6,8). They are moreover capable of removing calcium ions present in hydroxyapatite crystals (7-9), and they may change the structural composition of human dentin (9-13), adversely affecting the adhesion of materials such as resin-based cements and root canal sealers to root dentin (14-15).

The decalcifying efficacy of solutions such as EDTA, citric acid or phosphoric acid is known to depend on the concentration, pH, and time of application (11,16-19). Research into maleic acid (MA) as an irrigating solution holds interest in the light of several advantages. Compared to 17% EDTA, a 7% MA solution can prove more effective for smear layer removal from the apical third of instrumented root canals (20) it has a higher decalcifying activity (21), affords better post-obturation apical seal (22), is less cytotoxic (23) and it shows greater antimicrobial activity against *Enterococcus faecalis* biofilms (24,25) as well as equivalent activity against several endodontic microorganisms (26).

The combination of surfactant agents with antiseptics or chelating agents (MTAD, Tetraclean, Smear Clear, Cetrexidin, Chlor-XTRA, CHX-Plus) is recommended to reduce the surface tension of irrigants and facilitate their penetration into places of difficult access (27). Cetrimide (CTR) is a cationic surfactant that has shown, alone or associated with CHX (28), EDTA, and citric or maleic acids (24), to exert effective bactericidal activity against *E. faecalis* biofilms. However, no studies to date have assessed the decalcifying effects of the association of CTR with MA and CHX.

The aim of this study was to determine the decalcifying efficacy of 7% MA, 2% CHX, and combinations of 7% MA + 0.2% CTR, and 2% CHX + 0.2% CTR, in 1, 2, 3 and 5 minutes.

## Material and Methods

-Specimen preparation

The crowns of ten periodontal central superior incisors —stored in distilled water with thymol crystals until use were removed at the cementumenamel junction level using an Accutom-50 diamond cutter (Accutom Hard Tissue Microtome, Struers, Ballerup, Denmark) under water cooling. Patients were informed that the teeth would be used in this study, applying relevant ethical criteria. Root cementum was removed from the root surface using a fine-grained diamond bur (Perio-Set, Intensive. Grancia, Switzerland) at low speed under low water cooling.

One transversal section of 2-mm thickness was obtained from the coronal third of each root using an Accutom 50 automatic preprogrammed machine (Accutom Hard Tissue Microtome). Each slice was then divided into four equal portions, so as to obtain a total of four sections of each root (S1-S4). Sections were weighed on an A&D HM 202 precision balance (A&D Engineering Inc, San Jose, CA), equalizing their weight with disks of 600-grit silicon carbide paper (WS 18-B Struers, Ballerup, Denmark), which were always applied to the same surface to avoid altering the geometry of the disks. Sections were then labeled and stored in flasks with distilled water at room temperature until use. Sections of the same root (S1-S4) had approximately the same weight, geometry and degree of calcification, allowing for comparison of the decalcifying capacity of the different irrigating solutions by testing them on comparable specimens.

-Decalcifying procedures

The 40 sections obtained were divided into four experimental groups (n=10) for treatment with different combinations of irrigating solutions: 7% MA (pH 1.13), 2% CHX (pH 6.47), 7% MA + 0.2% CTR (pH 1.26) and 2% CHX + 0.2% CTR (pH 6.49). All chemical solutions were freshly prepared in laboratory conditions and were homogenized by constant stirring at 18-21 ºC using a magnetic multi-stirrer. The pH of each solution was determined using a pH-meter equipped with Micro PH 2000 electrode (Crisol, Alella, Spain). The accuracy of the pH-meter was ≤ 0.01.

Initially, 30 ml of each solution was prepared as a blank to determine calcium levels in the absence of specimen. Each sample was randomly immersed in 30 ml of irrigating solution for four immersion times (t1=1 min; t2=2 min; t3=3 min; t4=5 min). At the end of the time period, 5 ml of irrigation solution was extracted with a graduated pipette, then placed in hermetically sealed and labeled glass vessels.

-Spectrometer examination

Four extracts were obtained from each sample and measured in a SpectrAA 220 FS atomic absorption spectrometer (Varian Iberica SL, Madrid, Spain) using an air/acetylene mixture as fuel for the flame. The spectrometer was calibrated using solutions of 2, 5, and 10 ppm of Ca2+ as the reference pattern. The concentration of the original Ca2+ solution was 1000 ppm (Merck Inc., Whitehouse, NJ, USA). Extract readings were expressed in parts per million (ppm) and then transformed into mg of Ca2+ per gram (mg Ca2+/g) (17,18).

In a previous pilot study we demonstrated that distilled water and 0.2% CTR did not extract Ca2+ after a period of 5 minutes.

-Statistical analysis

Mean and standard deviations were determined for each group. The Shapiro-Wilk test was used to assess the distribution of the extracted calcium data. Because results for each group did not follow a normal distribution, variables were analyzed using the Mann-Whitney U-test (comparisons among solutions) and the Wilcoxon test (comparisons among times). The level of statistical significance was set at P<0.05. All statistical analyses were performed by means of SPSS 15.0 software (SPSS Inc, Chicago, IL).

## Results

The amounts of calcium extracted by each irrigant solution at the different immersion times are summarized in ( Table 1). Statistically significant differences were seen in the extracted calcium at all four time periods. The amount of calcium extracted by 7% MA was the highest at all immersion times, followed by 7% MA + 0.2% CTR. Two percent CHX and its combination with 0.2% CTR extracted virtually no calcium. When comparing the amount of calcium extracted by each solution at the four immersion times, statistically significant differences were seen except for 2% CHX and its combination with 0.2% CTR.

**Table 1:**
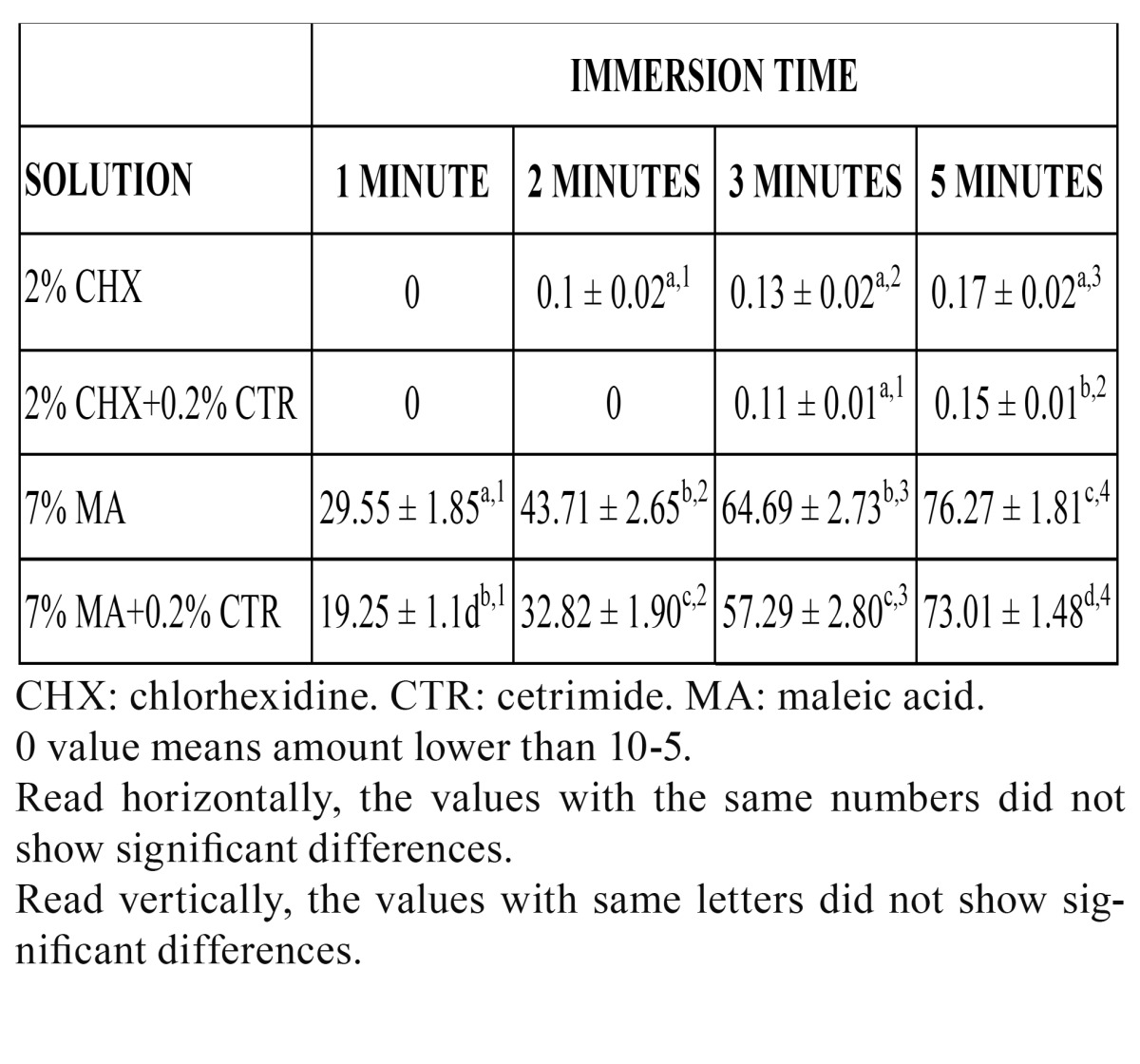
Amount of calcium extracted (mg Ca2+ x 10-3). Mean ± Standard deviation (n=10/group).

## Discussion

The effect of chemical agents used in endodontic treatment and their influence on the mechanical properties of root canal dentin have been investigated by various means, including microhardness measurements, micro-radiographic assessments and spectrometry studies (8-13,16-19). In the present study, experiments were conducted on sections obtained from the coronal third of root canals, uninstrumented (12), and having the same weight, geometry and calcification, thus allowing for comparison of the decalcifying capacity of the irrigating solution groups. We established time periods of 1 to 5 minutes, in line with most studies that indicate greater extraction of calcium ions of the root dentin at 3 minutes´ time (17,19) or at 5 minutes (16,18).

Our results indicate that the 2% CHX solution had an insignificant decalcifying effect on the root dentin, at all four time-periods. Although it is known that CHX removes only a small amount of calcium from the dentin in 5-15 minute intervals (12), the effect of its combination with CTR has not been described elsewhere to date. The behavior of CHX might be attributed to its cationic nature, meaning that this solution might exert an indirect effect on the removal of Ca2+ from root dentin (29) by binding anionic molecules present in the calcium carbonate complexes within the structure of hydroxyapatite (12). The bicationic nature of CHX would explain the small amount of calcium extracted by 2% CHX and the lack of calcium extraction by monocationic 0.2% CTR (data not published). Accordingly, the negligible extraction of calcium ions obtained with 2% CHX + 0.2% CTR would be advantageous for use as a final irrigant: aside from not altering the mineral content of the root dentin, recent studies point to its efficacy eradicating *E. faecalis* biofilms (28) as well as its residual antimicrobial activity in root canals infected with *E. faecalis* (30).

In the present study, 7% MA extracted an amount of calcium significantly greater than that obtained when combined with 0.2% CTR. For both solutions, the amount of calcium was seen to increase over the established time periods. It has been demonstrated that MA, used at a concentration ranging from 5% to 15%, effectively removes smear layer; but an increase in its concentration to 10% or more can result in demineralization and damage to the root canal wall (31). Recently, Ballal et al. obtained higher decalcification values with 7% MA than with 17% EDTA (21). In this context, the lesser decalcifying capacity shown in this study by 7% MA + 0.2% CTR as compared to 7% MA, in addition to the greater effectiveness than 17% EDTA in removing smear layer from the apical third of the root canal system (20) and its proven antimicrobial activity against *E. faecalis* biofilms (24,30), serve to reinforce the recommendation of 7% maleic solution as an effective alternative to the routine use of 17% EDTA (20).

The association of 0.2% CTR with 7% MA diminished the decalcification capacity of this solution, a finding that could be at-tributed to the slightly higher pH; accordingly, the rise in hydroxyl ions would hinder the ionic dissociation of hydroxyapatite, limiting the availability of Ca2+ ions (31). Sayin et al. (11) found that 17% EDTA extracted more Ca2+ ions than EDTAC (15% EDTA + 0.1% cationic surfactant, cetavlon [cetyltrimethyl ammonium bromide]) when used as a single irrigating solution, in a study evaluating calcium removal on root canal dentin at 1 and 5 minutes.

The diminished extraction of calcium ions from root dentin obtained with the association of CTR and MA would be beneficial during root canal preparation, helping maintain the structural composition of root canal dentin in alternating irrigation regimens (11,32). Furthermore, CTR reduces the surface tension of MA, facilitating its penetration of the root dentin at depth (27). The effectiveness of 7% MA + 0.2% CTR in eradicating* E. faecalis* biofilms in as little as 30 seconds has been demonstrated as well (24).

In sum, the context of the present study leads us to conclude that the calcium extracted from root dentin by 7% MA + 0.2% CTR is lower than that extracted when a solution of 7% MA is used.
